# Supplementary material for: Simplified Synthesis of Renieramycin T Derivatives to Target Cancer Stem Cells via β-Catenin Proteasomal Degradation in Human Lung Cancer
Source: Mar Drugs. 2023 Nov 30;21(12):627. doi: 10.3390/md21120627 (PMC10744608; doi:10.3390/md21120627)
Supplement: Supplementary file 1 [file marinedrugs-21-00627-s001.zip › marinedrugs-2744304-supplementary.pdf]

Supporting Information

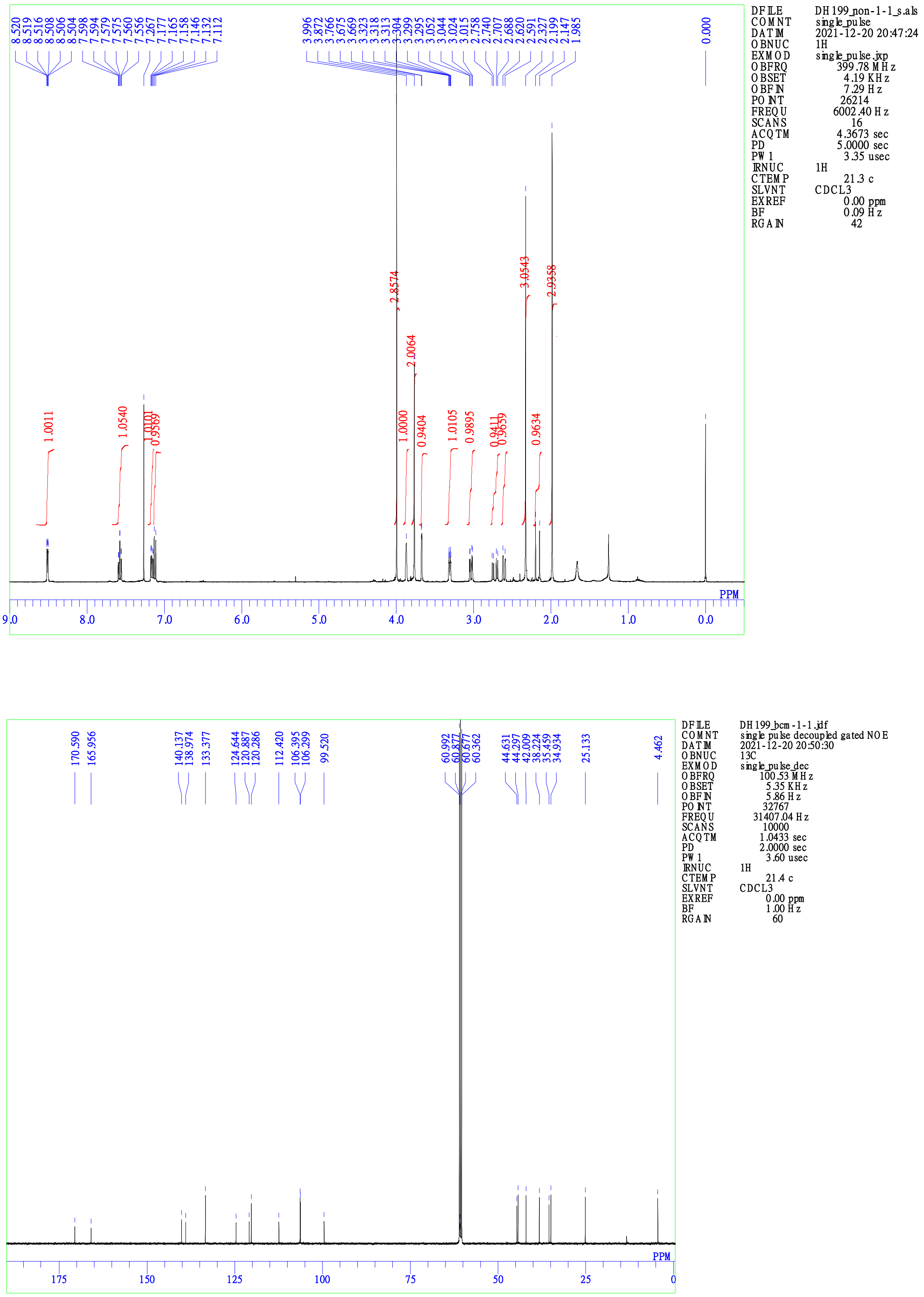

A

B

Figure S1. (**A**). ^{1}H-NMR of **4a: DH_18** in $CDCl_3$ (400 MHz). (**B**). ^{13}C-NMR of **4a: DH_18** in $CDCl_3$ (100 MHz).

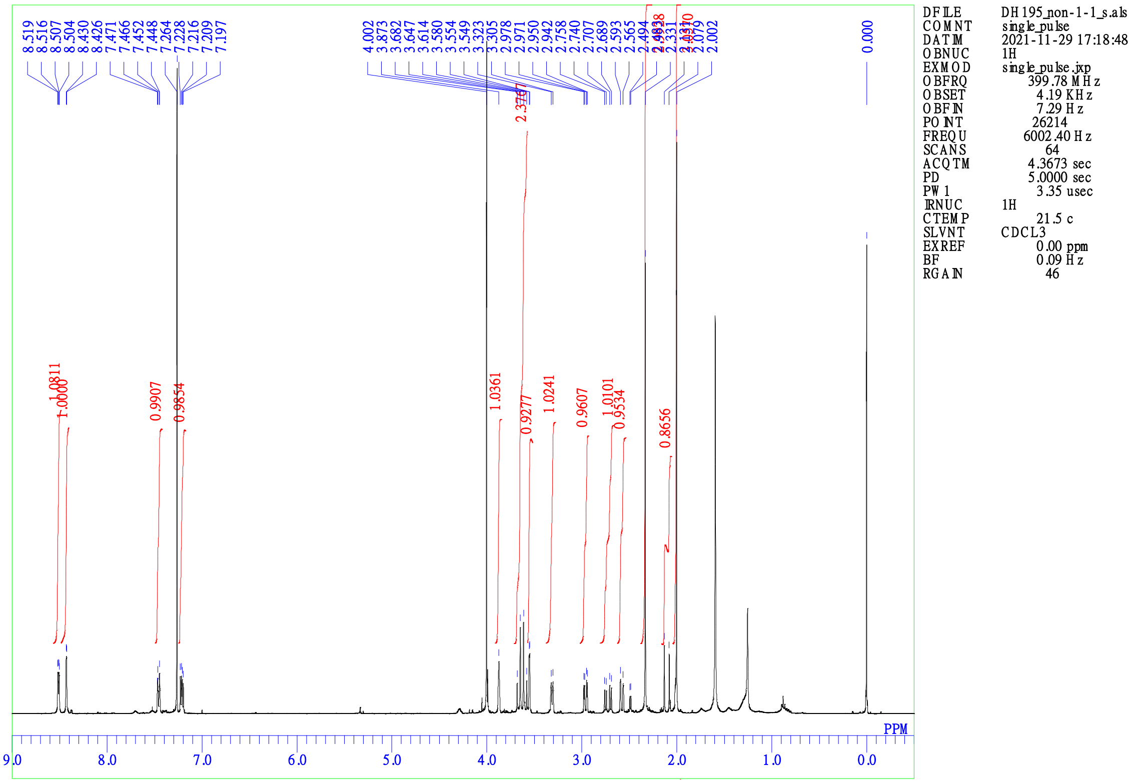

A

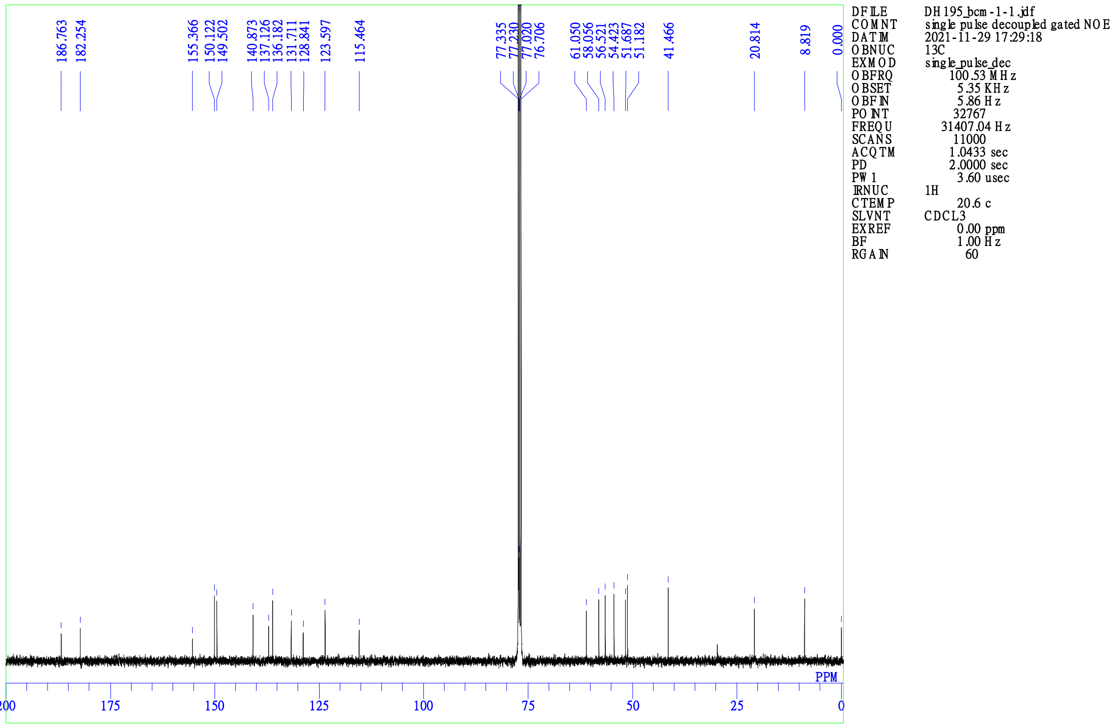

B

Figure S2. (**A**). ^{1}H-NMR of **4b: DH_21** in $CDCl_3$ (400 MHz). (**B**). ^{13}C-NMR of **4b: DH_21** in $CDCl_3$ (100 MHz).

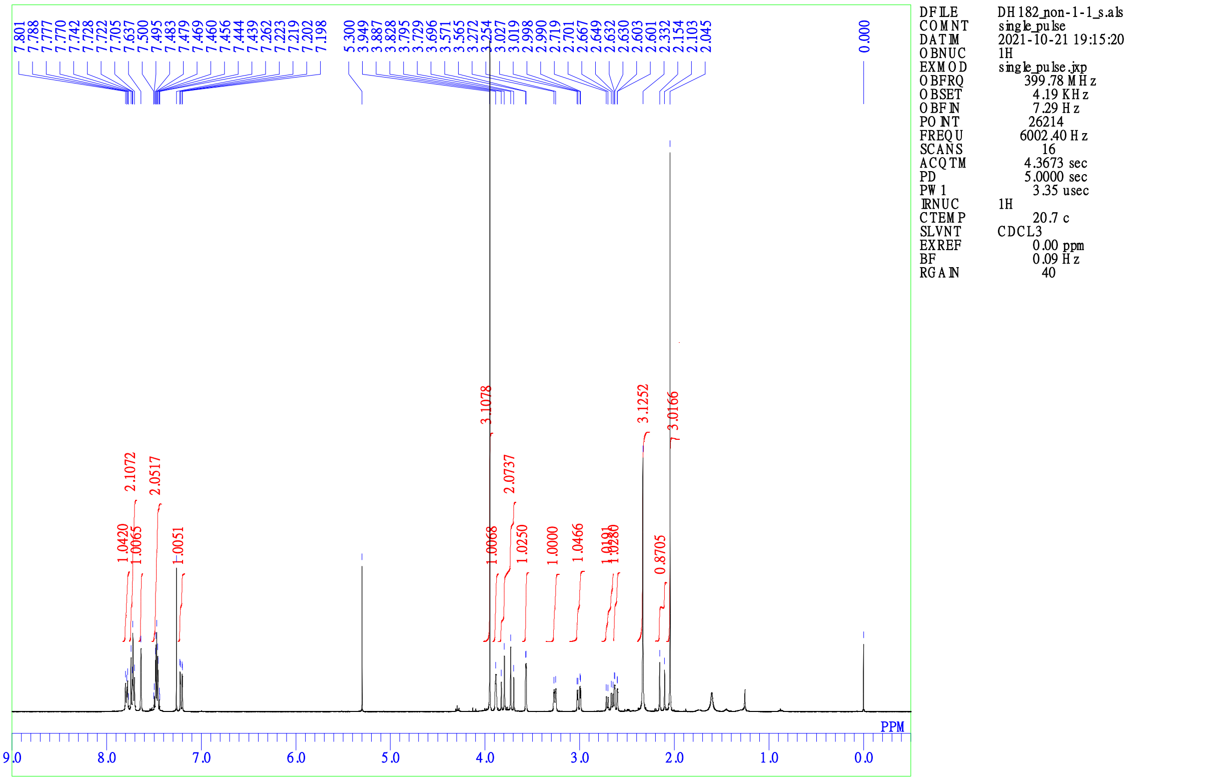

A

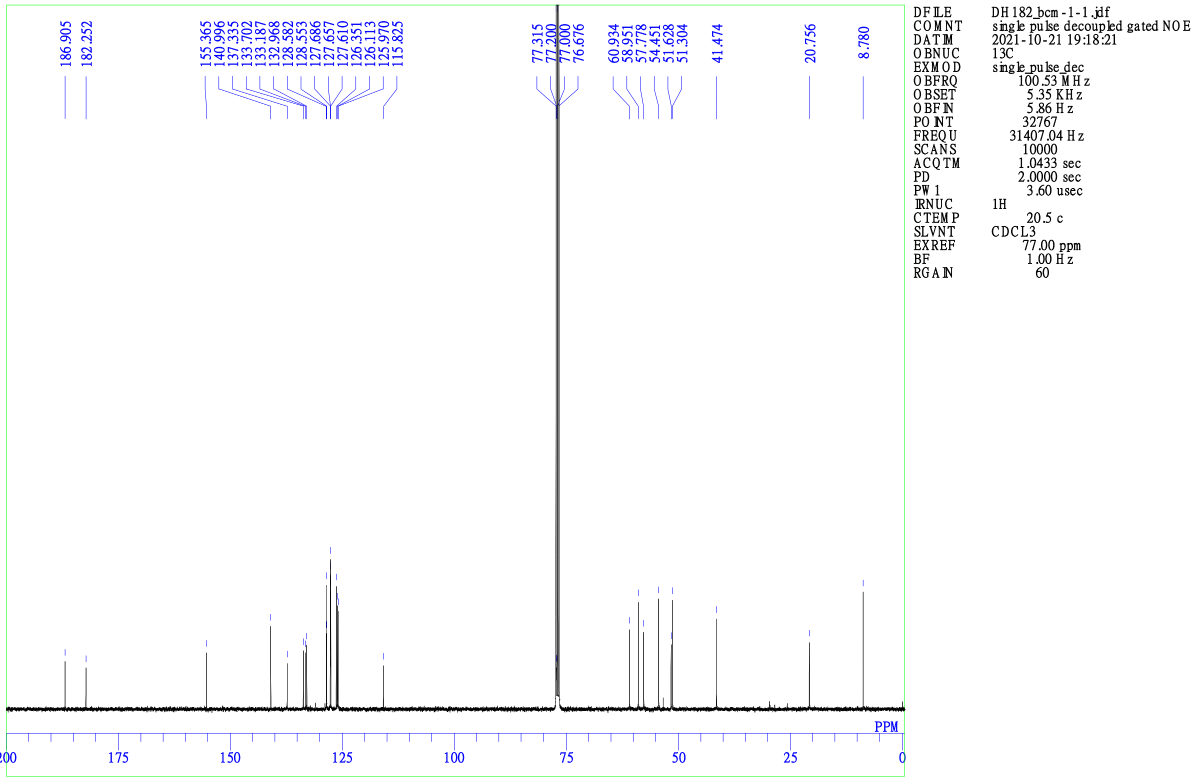

B

Figure S3. (**A**). ^{1}H-NMR of **4c: DH_32** in $CDCl_3$ (400 MHz). (**B**). ^{13}C-NMR of **4c: DH_32** in $CDCl_3$ (100 MHz).

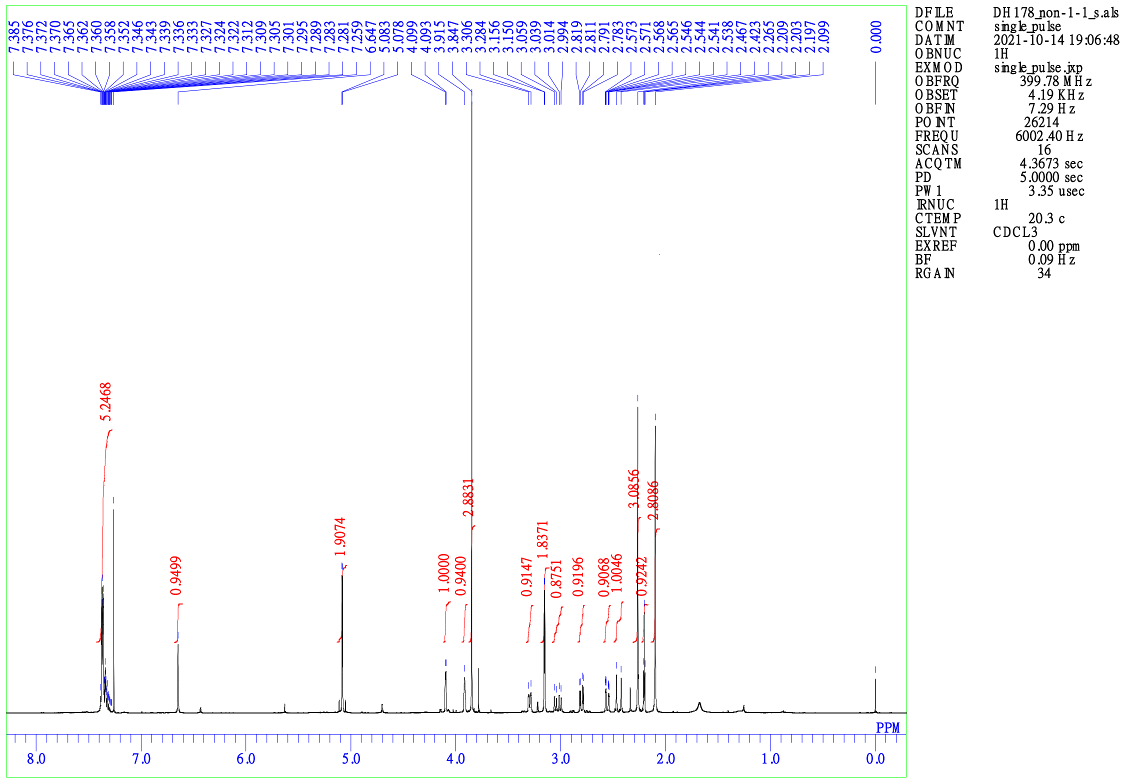

A

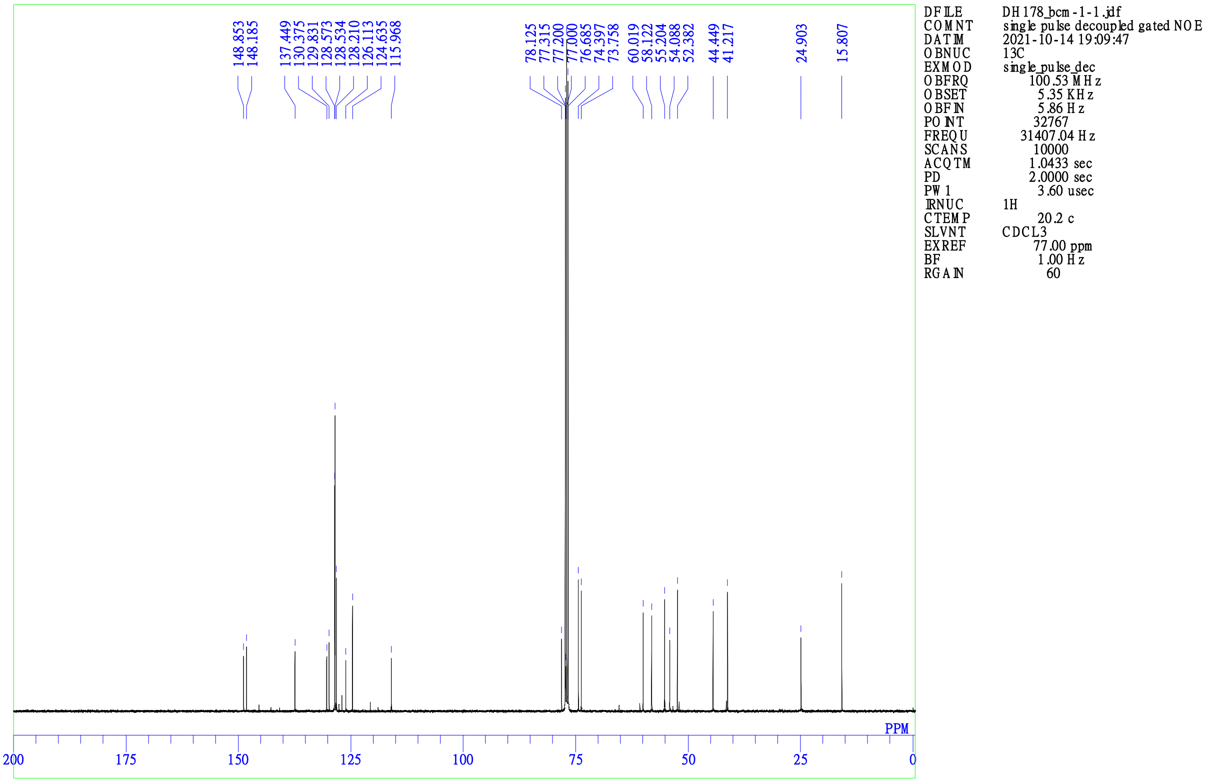

B

Figure S4. (**A**). ^{1}H-NMR of **2d** in $CDCl_3$ (400 MHz). (**B**). ^{13}C-NMR of **2d** in $CDCl_3$ (100 MHz).

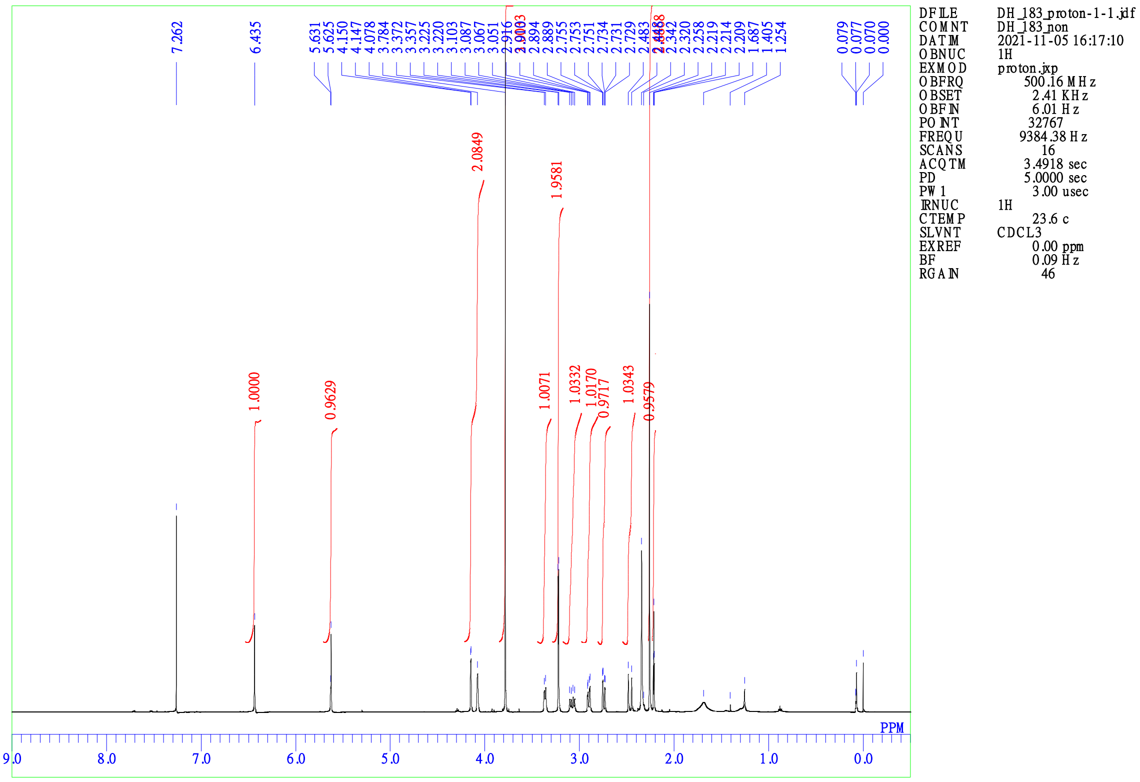

A

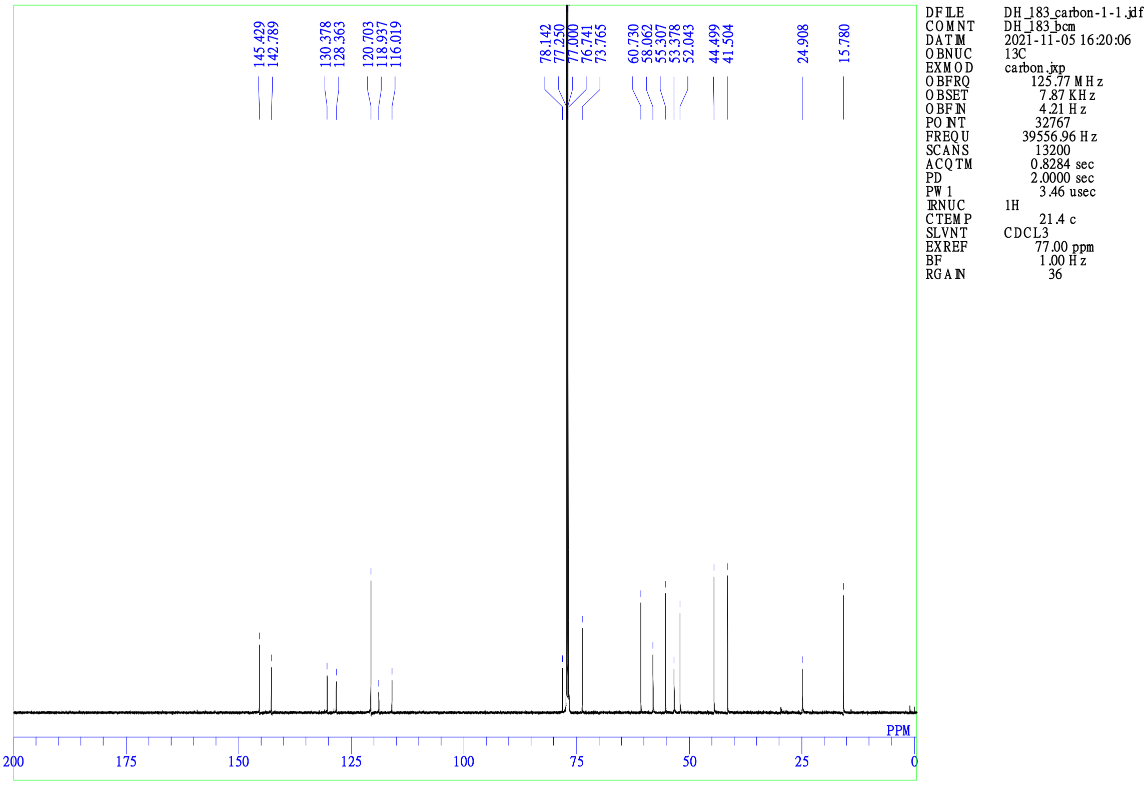

B

Figure S5. (**A**). ^{1}H-NMR of **3d** in $CDCl_3$ (500 MHz). (**B**). ^{13}C-NMR of **3d** in $CDCl_3$ (125 MHz).

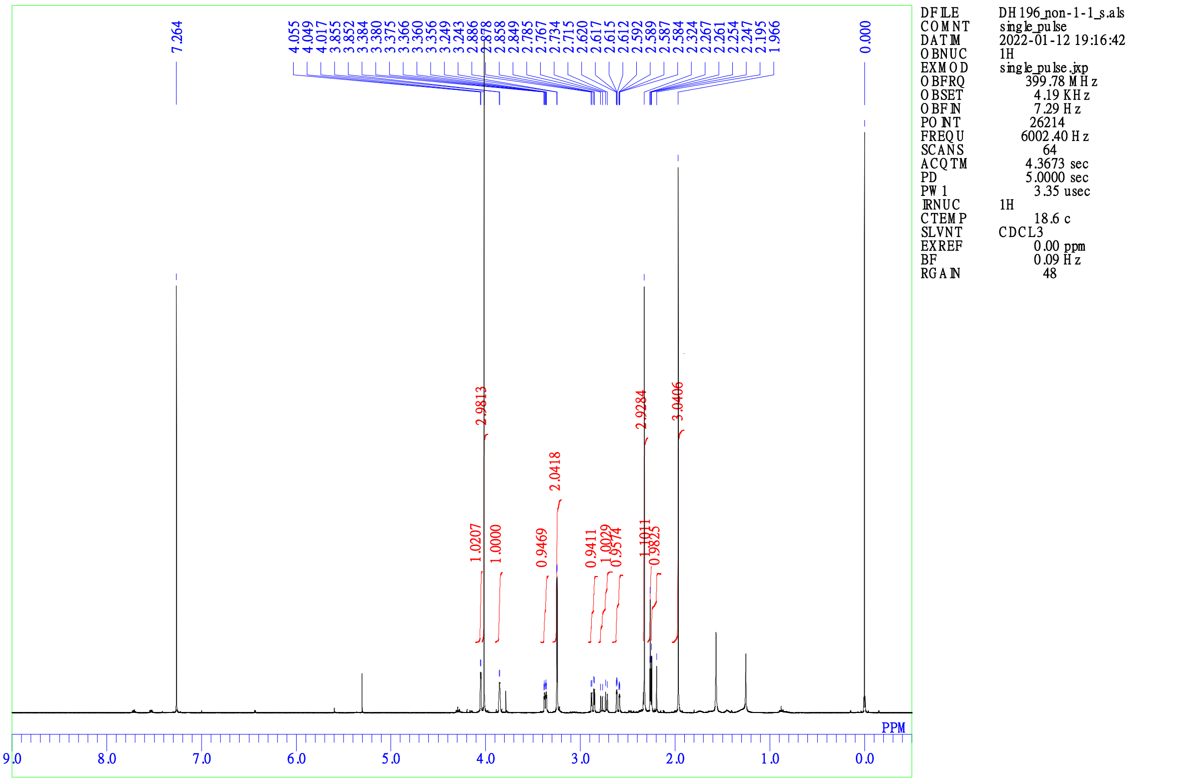

A

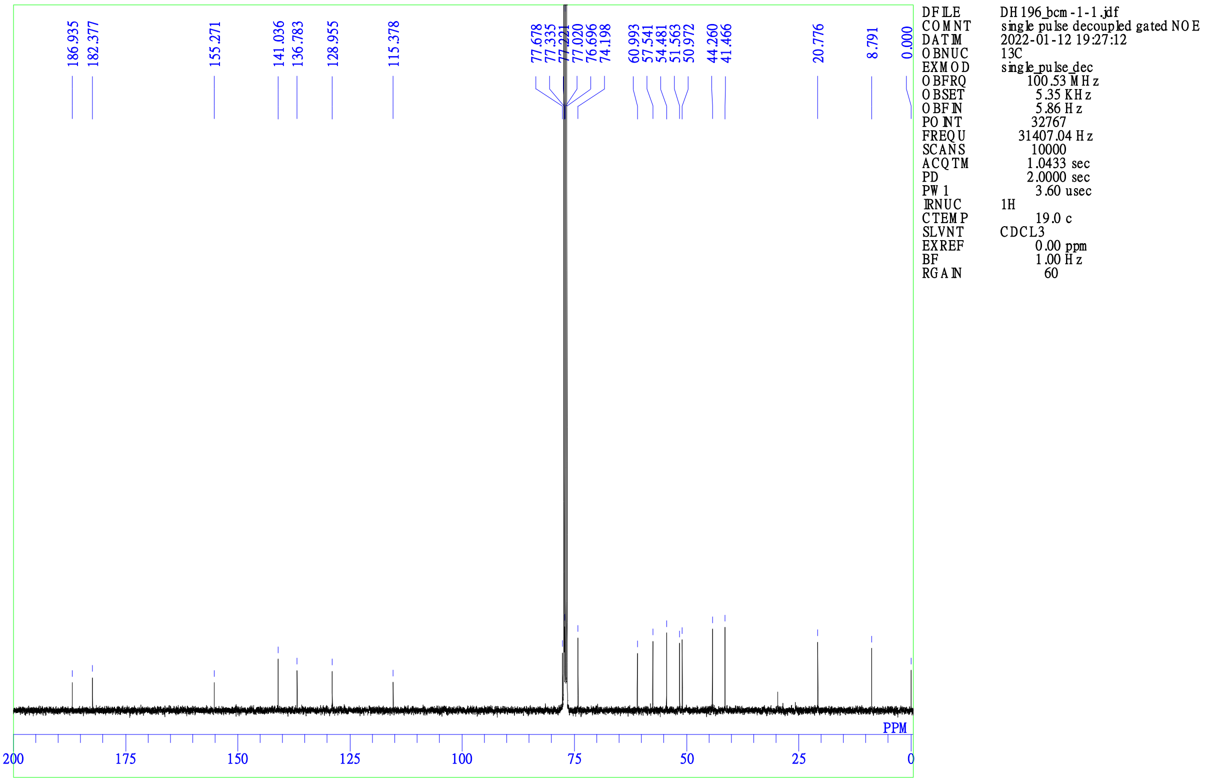

B

Figure S6. (**A**). ^{1}H-NMR of **4d: DH_35** in $CDCl_3$ (400 MHz). (**B**). ^{13}C-NMR of **4d: DH_35** in $CDCl_3$ (100 MHz).

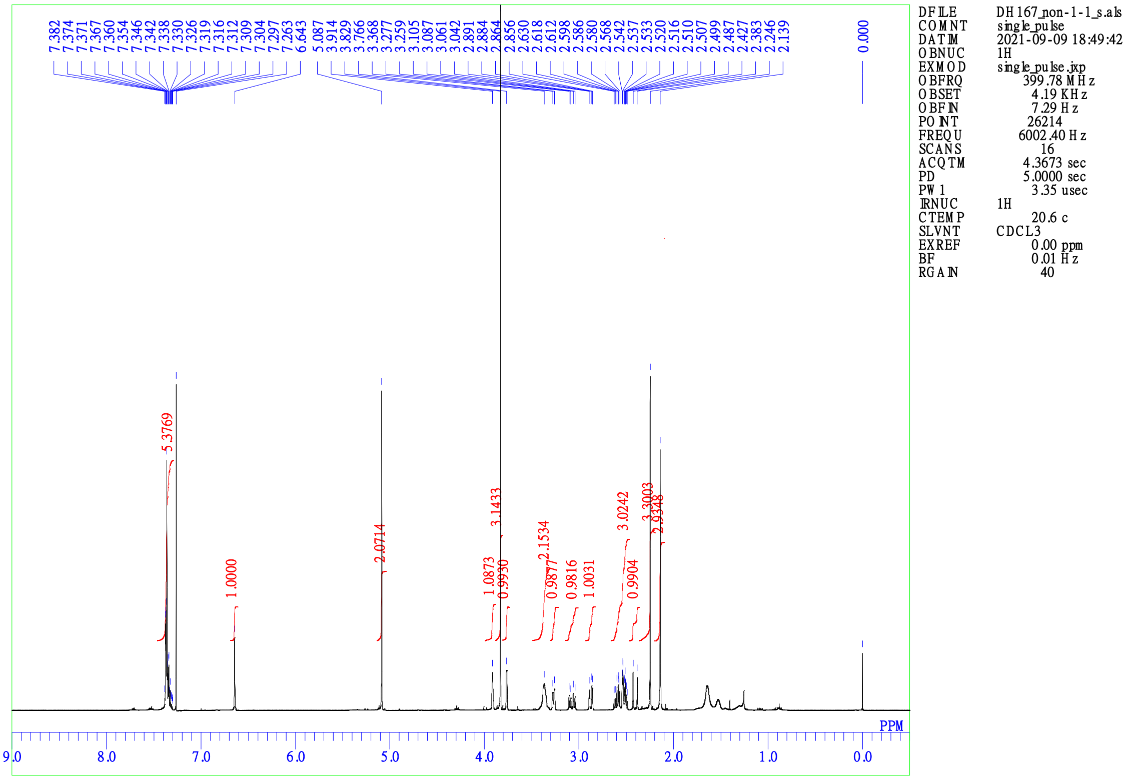

A

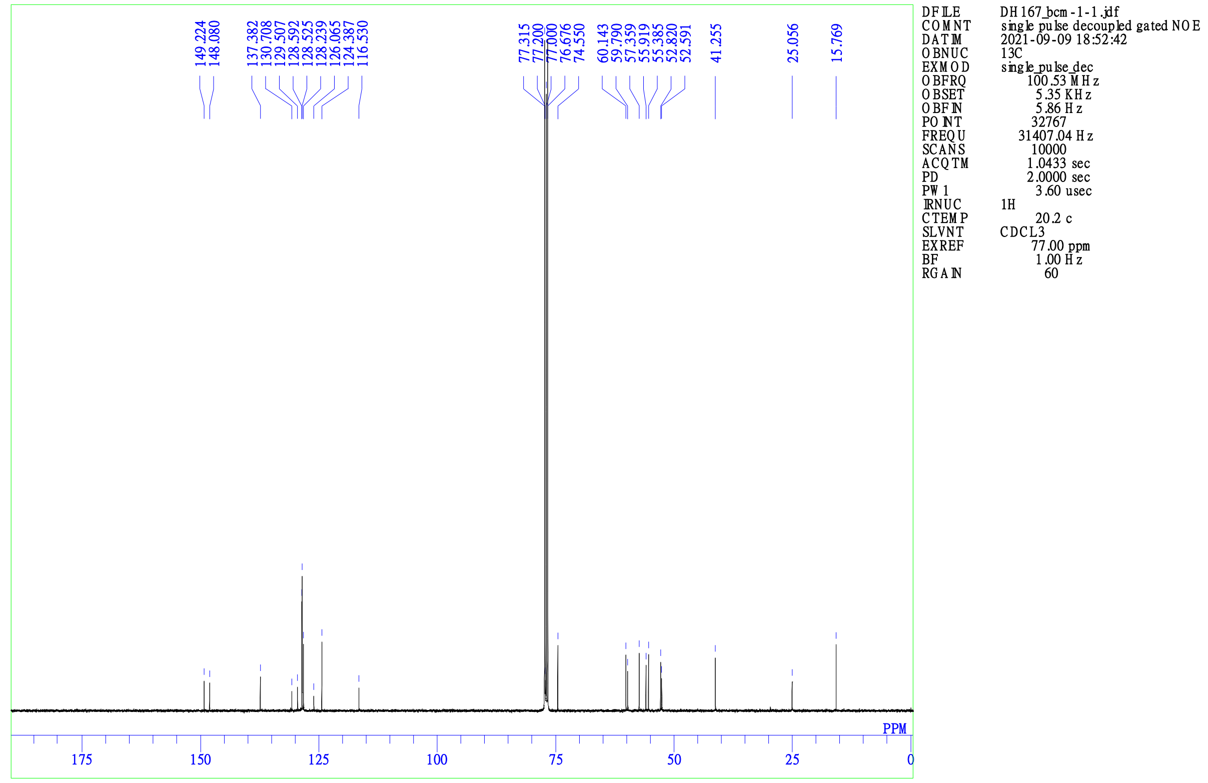

B

Figure S7. (**A**). ^{1}H-NMR of **2e** in $CDCl_3$ (400 MHz). (**B**). ^{13}C-NMR of **2e** in $CDCl_3$ (100 MHz).

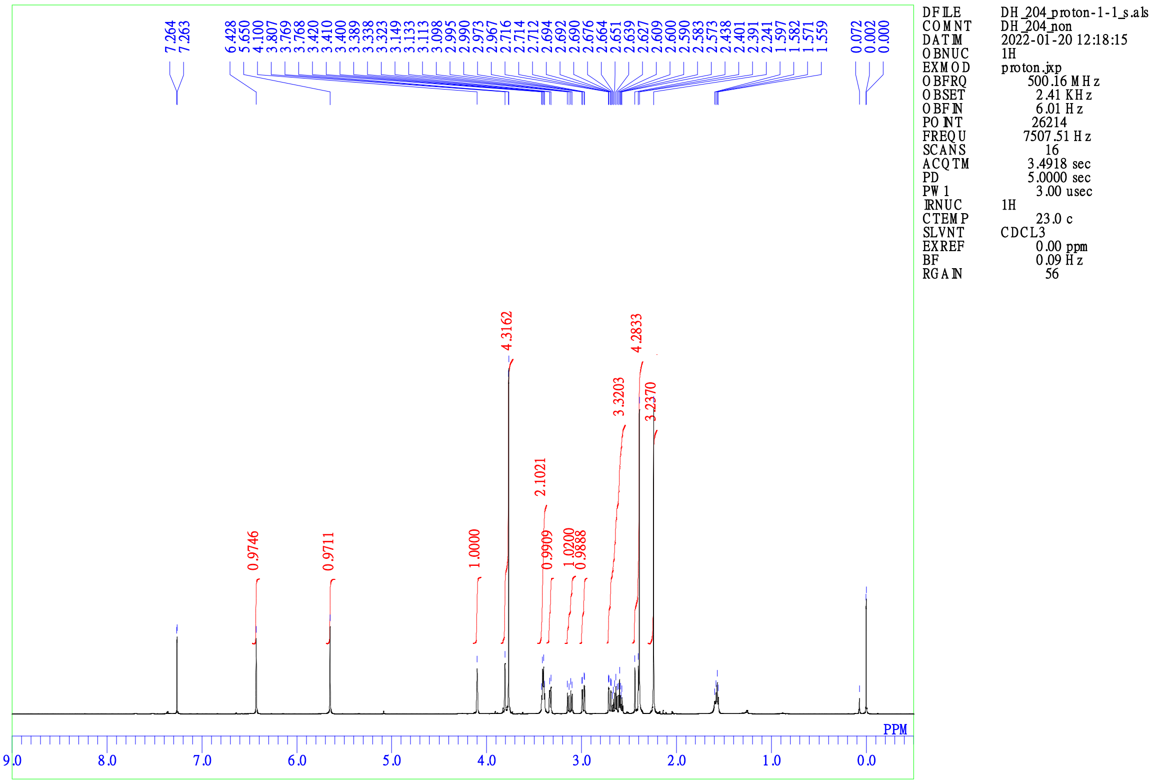

A

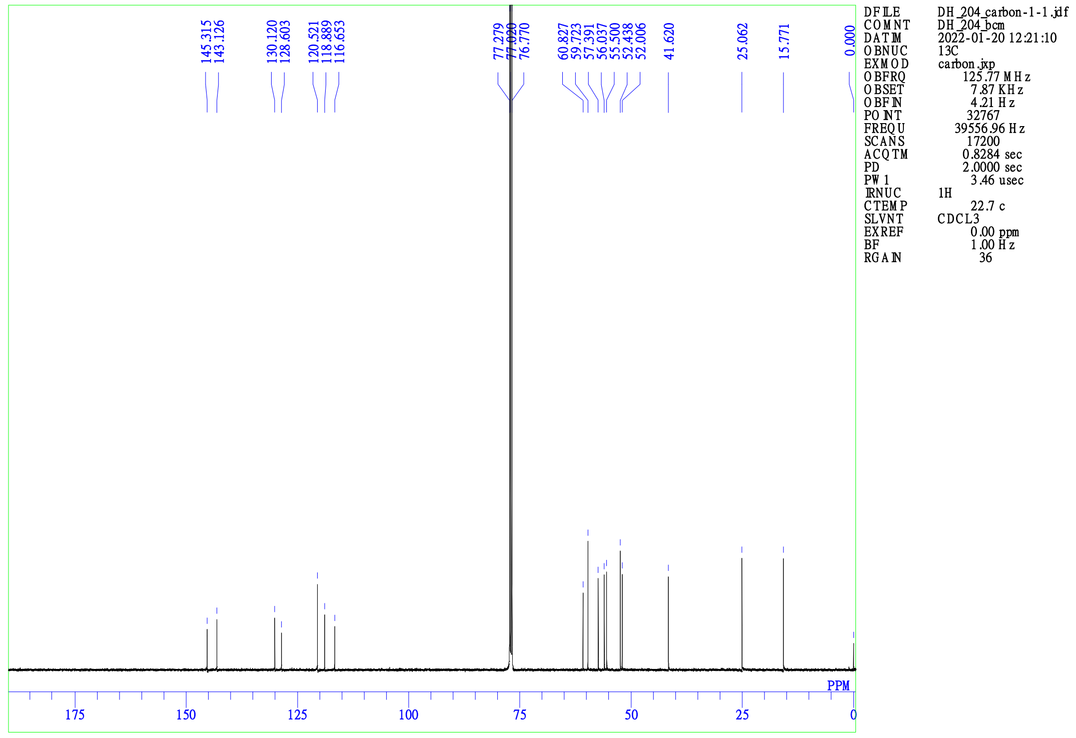

B

Figure S8. (**A**). ^{1}H-NMR of **3e** in $CDCl_3$ (500 MHz). (**B**). ^{13}C-NMR of **3e** in $CDCl_3$ (125 MHz).

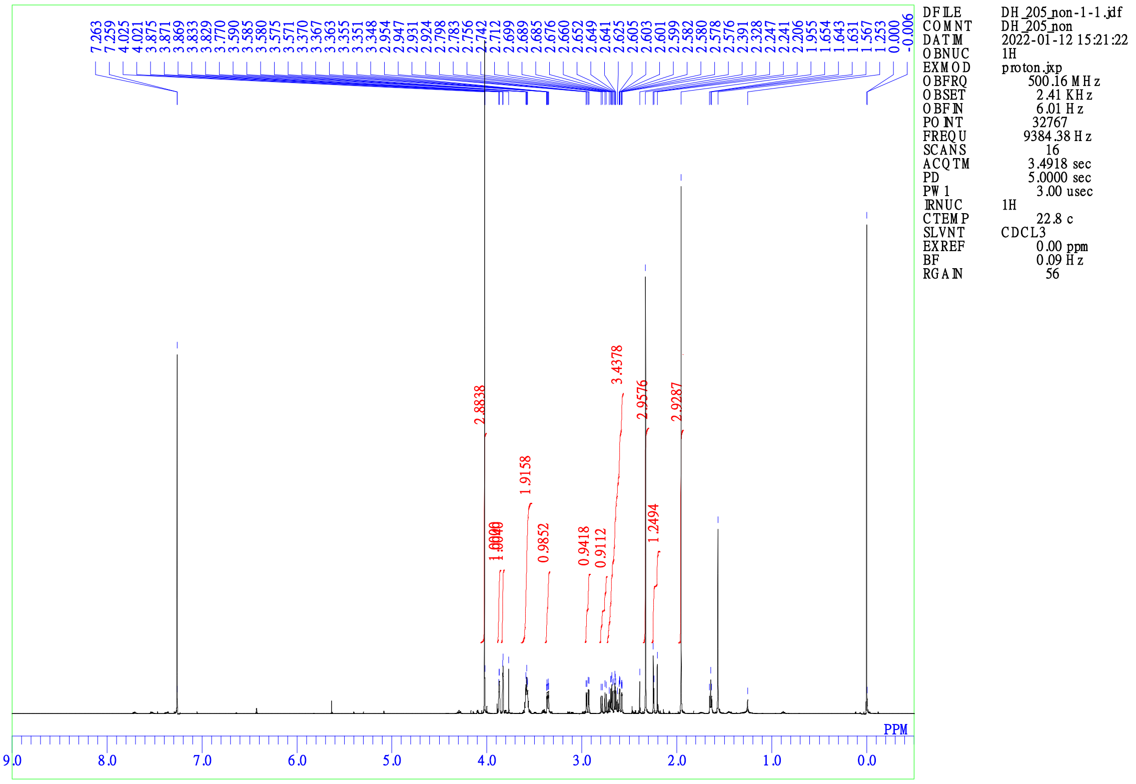

A

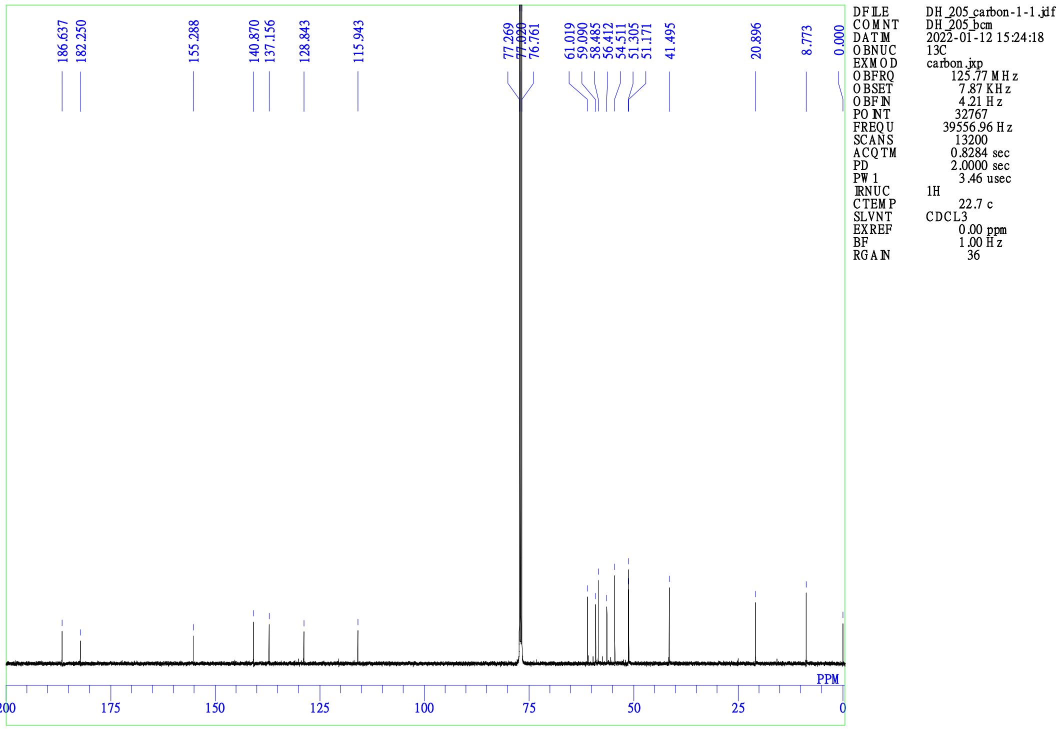

B

Figure S9. (**A**). ^{1}H-NMR of **4e: DH_38** in $CDCl_3$ (500 MHz). (**B**). ^{13}C-NMR of **4e: DH_38** in $CDCl_3$ (125 MHz).

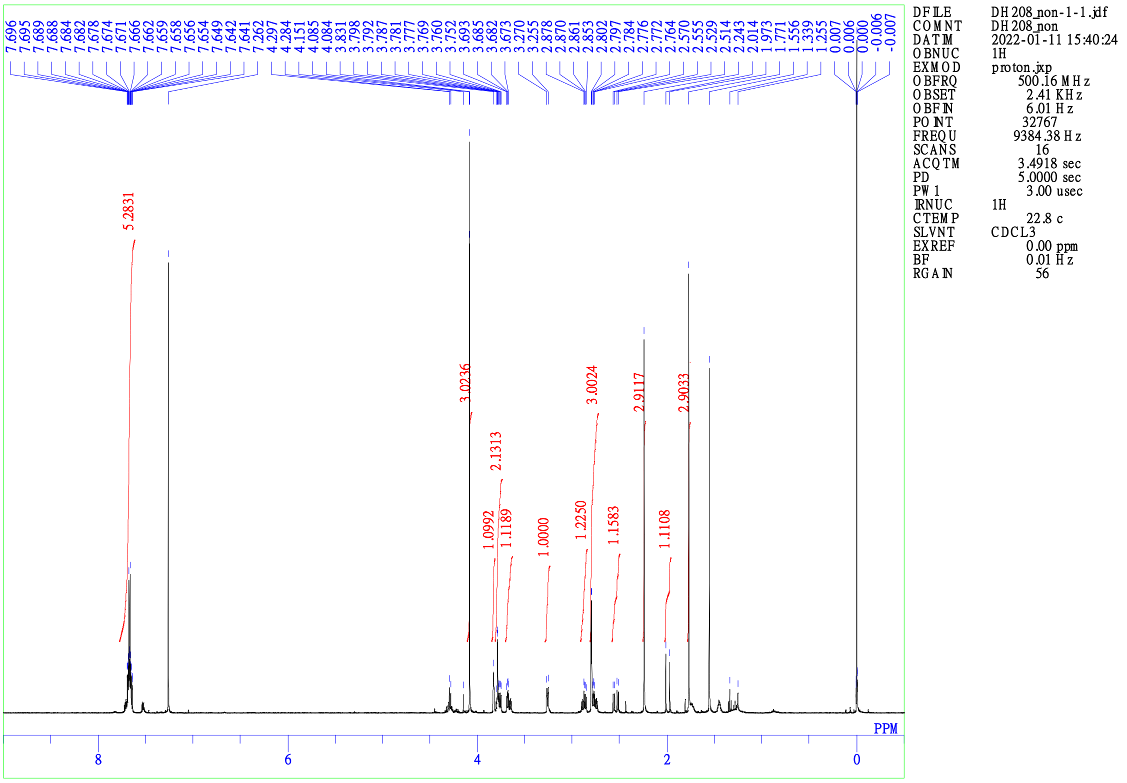

A

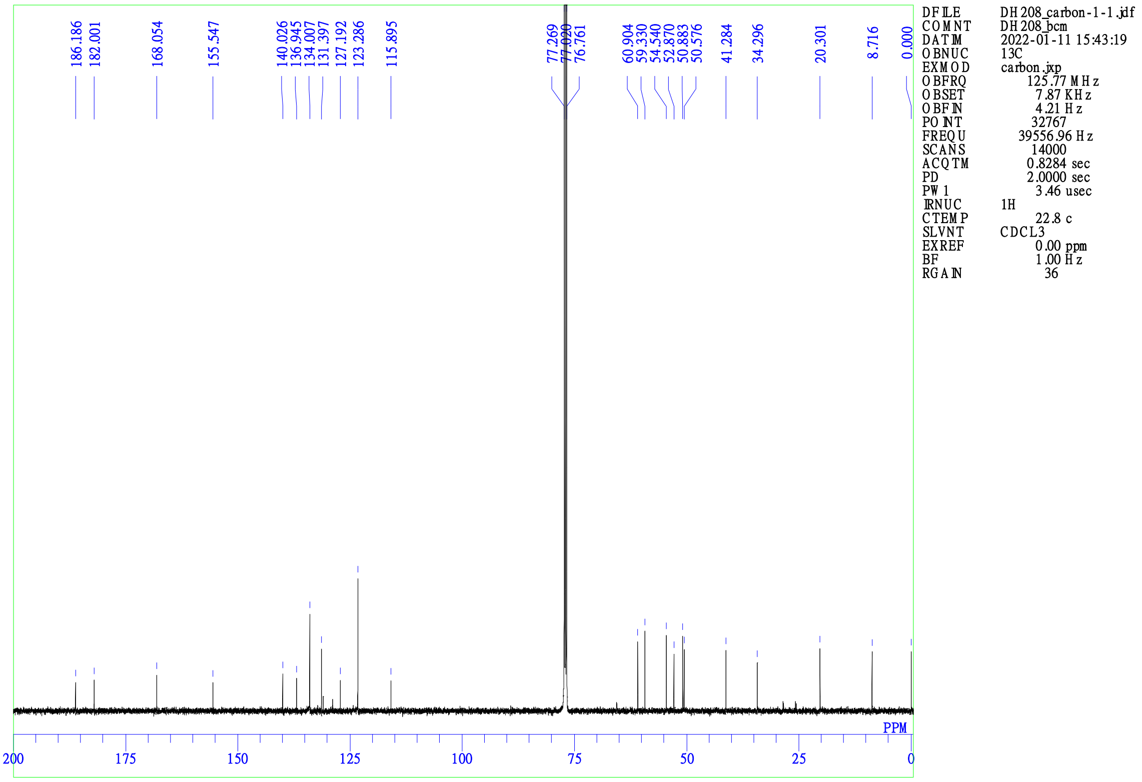

B

Figure S10. (**A**). ^{1}H-NMR of **4f: DH_39** in $CDCl_3$ (500 MHz). (**B**). ^{13}C-NMR of **4f: DH_39** in $CDCl_3$ (125 MHz).
